# Understanding the implementation of antimicrobial stewardship in Utah community pharmacies

**DOI:** 10.1093/jacamr/dlaf155

**Published:** 2025-09-03

**Authors:** Tariq A Mosleh, April Clements, Devin Beard, Angela Weil, Christina Radloff, Charisse Schenk, Sarah Rigby, Susan Cheever

**Affiliations:** Utah Department of Health and Human Services, 288 N 1460 W, Salt Lake City, UT 84116, USA; Utah Department of Health and Human Services, 288 N 1460 W, Salt Lake City, UT 84116, USA; Utah Department of Health and Human Services, 288 N 1460 W, Salt Lake City, UT 84116, USA; Utah Department of Health and Human Services, 288 N 1460 W, Salt Lake City, UT 84116, USA; Utah Department of Health and Human Services, 288 N 1460 W, Salt Lake City, UT 84116, USA; Utah Department of Health and Human Services, 288 N 1460 W, Salt Lake City, UT 84116, USA; Utah Department of Health and Human Services, 288 N 1460 W, Salt Lake City, UT 84116, USA; Utah Department of Health and Human Services, 288 N 1460 W, Salt Lake City, UT 84116, USA

## Abstract

**Objectives:**

Antimicrobial stewardship (AS) is essential to optimize antibiotic use and combat resistance. This study evaluated the implementation of AS activities in community pharmacies across Utah and assessed pharmacists’ understanding of AS principles, current practices and barriers. This study also aimed to increase community pharmacists’ awareness of preventing drug-resistant infections. Addressing community pharmacies AS barriers can significantly enhance AS efforts, to ensure optimal patient outcomes while combating antibiotic resistance.

**Methods:**

A cross-sectional survey was distributed to community pharmacies throughout Utah. The survey collected data on pharmacy demographics, AS knowledge, implementation practices, perceived barriers and potential facilitators related to AS.

**Results:**

Out of 110 respondents, 50% worked in privately owned pharmacies, and 78.7% held a Doctorate in Pharmacy. While 71.6% were familiar with ASP, 28.4% were not. Major barriers to implementing AS in community pharmacies included limited access to patient data (79.8%) and the absence of standardized AS guidelines (53%). Pharmacists identified several key facilitators that could support AS implementation, which included access to guidelines (75.2%) and better collaboration with providers (75.2%). Additionally, (86.4%) of pharmacists highlighted improved access to patient records was essential or effective for AS implementation. However, only (47.7%) of pharmacists consistently intervened when an antibiotic dosage or frequency appeared inappropriate, which indicates potential gaps in AS-related interventions.

**Conclusions:**

Enhancing pharmacists’ access to clinical data and fostering collaboration with healthcare providers are crucial steps towards effective AS implementation in community settings.

## Introduction

Antibiotic resistance is a major public health threat, contributing to 2.8 million drug-resistant infections and 35 000 deaths annually in the USA.^1^ Resistant infections often require more expensive alternatives with higher side effects, which increase healthcare costs, morbidity and mortality.^1^

Inappropriate antibiotic prescribing is known to contribute to the increase in antibiotic resistance; it is estimated that up to 50% of outpatient prescriptions are inappropriate^2,3,4^ and up to 30% could be unnecessary.^5^ Outpatient settings account for 60% of US antibiotic expenditures,^6,7^ with pharmacies dispensing ∼236.4 million prescriptions in 2022 alone.^8^

Antimicrobial stewardship (AS) optimizes antibiotic use by ensuring appropriate prescribing and minimizing misuse.^9^ Given that 20% of pediatric and 10% of adult outpatient visits result in antibiotic prescriptions,^3,10^ community pharmacies present a critical opportunity for stewardship interventions. Inappropriate use also contributes to adverse effects, leading to 143 000 emergency visits annually^11^ and increasing the risk of *Clostridioides difficile* infection.^12^

Implementing AS in US community pharmacies is crucial to combat antimicrobial resistance infections.^1^ Community pharmacists can play a vital role in promoting responsible antibiotic use, educating patients on proper medication adherence and ensuring appropriate prescribing practices.^13,14^ Community pharmacists can improve the appropriateness of antibiotics dispensed to their patients and enhance patient satisfaction by preventing side effects. Verification of a patient’s allergies will also improve antibiotic appropriateness.

Community pharmacists can contribute to reducing antibiotic resistance infections, through optimizing therapy and preventing harm to patients. Community pharmacists can be the last gate to optimize therapy through communication directly with both physicians and patients.^14,15^ Allowing community pharmacists to use their clinical knowledge and connections with their patients can have a huge impact on AS. A minute or two of a trusted pharmacist’s time spent with their patients who receive antimicrobials can raise awareness of this issue and help combat multidrug resistance.

Antimicrobial resistance poses a significant public health challenge, leading to increased morbidity, mortality and healthcare costs. AS aim to optimize antibiotic use, ensuring effective treatments and minimizing resistance. Community pharmacists play a crucial role in these programmes due to their accessibility and frequent patient interactions. The purpose of this study is to evaluate how well community pharmacists in Utah understand and implement AS activity (ASA) and identify barriers and potential solutions.

## Methods

A cross-sectional survey was conducted among community pharmacists in Utah utilizing the Research Electronic Data Capture (REDCap) to distribute the survey, based on Centers for Disease Control and Prevention core elements and reviewed by experts for clarity and relevance. The questionnaire encompassed demographics, knowledge of ASA, current practices, perceived barriers and facilitators for ASA implementation. Descriptive statistics were used to analyse the data (Appendix S1, available as Supplementary data at *JAC-AMR* Online).

### Recruitment

The survey was emailed to pharmacy managers at 321 Utah community pharmacies in early summer 2023 with a 120 day completion period. The email list was obtained from the Utah Division of Professional Licensing, Board of Pharmacy, and the survey was sent to the registered email addresses of pharmacy managers to ensure that responses were obtained from individuals in a leadership role within each community pharmacy. A reminder email was sent weekly until the closing date.

### Data analysis

Survey responses were downloaded from REDCap into a csv file. Survey excel file data were analysed using descriptive statistics.

## Results

This study surveyed 321 pharmacy managers across Utah, with a response rate of 34.3% (*n* = 110). The majority of respondents worked in private pharmacies (50%), followed by chain pharmacies (39.8%), outpatient hospital settings (5.9%) and inpatient hospital pharmacies (2.5%). Most respondents (78.7%) held a Doctorate in Pharmacy, with 18.5% having a bachelor’s degree, indicating a highly educated cohort. The majority of pharmacists were located in metropolitan areas (62%), while (38%) practiced in rural settings (Table 1).

**Table 1.** Community pharmacists’ demographic and knowledge of AS

Pharmacy demographics and pharmacist knowledge	%
Type of pharmacy	
Private pharmacy	50
Chain pharmacy	39.8
Outpatient hospital	5.9
Inpatient hospital	2.5
Other	1.7
Highest level of pharmacy education	
Doctorate degree	78.7
Bachelor’s degree	18.5
Master’s degree	3.4
Other	0
Work location	
Metro	62
Rural	38
Remote	0
Familiar with ASAs before the survey	
Yes	71.6
No	28.4
Understanding of ASAs after reading the definition	
A lot better	21.3
A bit better	40.7
Already knew	38
Still unsure	0

After providing a standardized AS definition, 40.7% reported a slight improvement in their understanding, and 21.3% reported significant improvement which suggests that structured education could further enhance pharmacists’ AS knowledge (Table 1).

Community pharmacists reported high levels of patient engagement regarding antimicrobial use. However, fewer pharmacists (47.7%) consistently intervene when an antibiotic dosage or frequency appears inappropriate, which indicates potential gaps in AS-related interventions.

Pharmacists identified several challenges in implementing AS. The most significant barrier was limited access to patient data, with 79.8% stating they lack the clinical or laboratory information necessary to review antimicrobial prescriptions effectively. Time constraints were also a concern, with 33.9% of respondents reporting insufficient time to fully engage in ASAs. Additionally, 41.5% of pharmacists indicated that prescribers were resistant to pharmacist-led interventions in antibiotic selection (Table 2).

**Table 2.** Barriers and facilitators to AS implementation

Identified barriers and facilitators		%
Barriers	
Limited access to patient data	79.8
No standard guidelines	53.4
Medical provider resistance	41.5
Time constraints	33.9
Lack of training	25.4
Facilitators	
Access to patient data	86.4
Access to guidelines	75.2
Better collaboration with providers	75.2
Compensation	69.5
Clarification of roles	55.9
Education programmes	52.5
Public awareness	46.6

Pharmacists identified several key facilitators that could support ASA implementation to address these barriers. More than half (52.5%) of respondents expressed a need for expanded educational programmes, such as workshops and webinars, to enhance AS-related knowledge and competencies. The provision of standardized guidelines for community infections was also emphasized, with 75.2% of pharmacists indicating that access to such guidelines would be highly beneficial (Table 2).

Interdisciplinary collaboration was another priority, with 75.2% of respondents advocating for better communication and cooperation with prescribers. Given the reported resistance from medical providers, fostering a more collaborative approach to antibiotic prescribing could enhance AS efforts in community settings. Additionally, 69.5% of pharmacists supported financial compensation for ASAs, suggesting that time constraints could be mitigated if pharmacists were reimbursed for their involvement in AS.

Finally, 86.4% of pharmacists highlighted improved access to patient records as a critical need (Table 2).

## Discussion

After providing AS definition, 62% reported an improved understanding, which suggests that education remains a critical component in enhancing pharmacists’ role in AS. This finding aligns with prior research emphasizing the need for continuous AS education among healthcare professionals, especially in community settings.^14,16^

Several barriers were identified which limit community pharmacists’ ability to engage in ASA. The most significant challenge for community pharmacists was limited access to patient data to review clinical and laboratory information and provide informed antimicrobial recommendations. The inability to assess diagnostic information restricts community pharmacists’ capacity to evaluate treatment appropriateness, potentially leading to unchecked inappropriate prescribing. Similar studies have identified this barrier as a common challenge for pharmacists who work outside hospital-based.^17,18^

Time constraints were a significant concern, indicating that lack of time hindered AS participation. Given the workflow demands in community pharmacies, AS responsibilities may not be prioritized, especially when pharmacists are managing high prescription volumes. Provider resistance to pharmacist interventions further complicates AS efforts, as reluctance from prescribers to accept pharmacist recommendations can discourage stewardship engagement. These findings are consistent with previous reports that emphasize the need for stronger pharmacist/prescriber collaboration in outpatient ASA.^19^

Pharmacists identified several key facilitators to strengthen AS in community pharmacies, including access to standardized guidelines, improved collaboration with prescribers and financial compensation for stewardship activities. Additionally, many emphasized the importance of access to patient records as the most critical intervention. Integrating pharmacists into electronic health record systems would support more effective AS interventions by enabling medication review and dose optimization using clinical data.

These findings reinforce the critical role of community pharmacists in AS but also highlight the structural limitations that restrict their full engagement. Policymakers and professional organizations should prioritize improving pharmacist access to patient information, standardizing AS protocols for community settings and developing reimbursement strategies to support pharmacists’ expanding role in AS.

Efforts to enhance inter-professional collaboration and provide targeted AS training could further strengthen community based AS initiatives. Given the high level of pharmacist engagement in patient education and intervention when barriers are minimized, optimizing the AS framework in community pharmacies could significantly improve antimicrobial use and resistance management at the population level.

A major strength of this study is the relatively high response rate among community pharmacy managers across Utah. The survey captured a diverse range of pharmacy settings, including private, chain and hospital outpatient pharmacies, enhancing the relevance of findings across different practice environments. However, the study is limited by its focus on pharmacists from a single US state, which may affect the generalizability of the results to other regions. Additionally, the use of self-reported survey data may introduce response bias.

### Conclusions

Despite high levels of AS awareness among pharmacists, nearly 30% of respondents lacked prior familiarity with AS principles, which highlights the need for additional training and educational initiatives. Pharmacists expressed strong willingness to participate in AS, recognizing its potential to reduce healthcare costs, enhance job satisfaction and elevate their role in antimicrobial management. However, without systematic improvements in interdisciplinary communication, the development of AS-specific guidelines for community pharmacies, and the integration of pharmacists into patient data systems, AS effectiveness will remain limited.

Findings from this study can inform statewide efforts to promote interdisciplinary collaboration among clinics, hospitals and pharmacies. This shift can move community pharmacy practice in Utah and beyond away from isolated operations towards integrated stewardship aligned with broader healthcare systems. Strengthening AS in community pharmacies through targeted interventions, addressing key barriers and leveraging pharmacists’ accessibility and expertise can significantly enhance AS impact and improve patient outcomes.

## Supplementary Material

dlaf155_Supplementary_Data

## Supplementary data

Appendix S1 is available as Supplementary data at *JAC-AMR* Online.
