# Supplementary material for: Understanding the implementation of antimicrobial stewardship in Utah community pharmacies
Source: JAC Antimicrob Resist. 2025 Sep 3;7(5):dlaf155. doi: 10.1093/jacamr/dlaf155 (PMC12406779; doi:10.1093/jacamr/dlaf155)
Supplement: dlaf155_Supplementary_Data [file dlaf155_supplementary_data.pdf]

Community pharmacist understanding and implementation of Antimicrobial Stewardship Program (ASP) in UTAH

Dear Sir or Madam,

Utah Department of Health and Human Services (DHHS) is reaching out to you to request your participation in a short survey. The purpose of this survey is to evaluate community pharmacist understanding and implementation of Antimicrobial Stewardship Program (ASP) in UTAH. Your participation is greatly appreciated and your responses will be kept confidential. Responses will be aggregated and summarized to protect anonymity. Thank you for taking the time to complete this survey. If you have any questions please contact Tariq Mosleh at tmosleh@Utah.gov

Sincerely,

Tariq Mosleh PharmD, PhD

HAI / AR Stewardship Pharmacist

Utah Department of Health and Human Services

tmosleh@Utah.gov

c: 801-718-3519

www.dhhs.utah.gov

The goals of this survey are to assess and measure community pharmacist's knowledge of Antimicrobial Stewardship Programs (ASP), to improve implementation of ASP practices in community pharmacies, and to provide educational feedback and materials on ASP to community pharmacists.

---

1) When did you graduate from pharmacy school? ____________________

2) How long has it been since you received continuing education concerning antibiotics? ____________________

3) Years of experience as a community pharmacist? ____________________

4) What type of pharmacy do you work at?
- ☐ Chain pharmacy
- ☐ Private pharmacy
- ☐ Out-patient hospital pharmacy
- ☐ In-patient hospital pharmacy
- ☐ Other

5) What is the highest level of pharmacy academic study achieved?
- ☐ Bachelor's Degree in Pharmacy
- ☐ Master's Degree in Pharmacy
- ☐ Doctorate Degree in Pharmacy
- ☐ Other

6) Main geographical location of work:
- ☐ Metro
- ☐ Rural
- ☐ Remote

7) If you also work in a sector of pharmacy other than community, please indicate this below.
- ☐ Hospital
- ☐ Industry
- ☐ Academia
- ☐ Others
- ☐ none

8)	I know the term Antimicrobial Stewardship Program (ASP)	◯ Yes<br>◯ No
9)	Antimicrobial Stewardship Program (ASP) is defined as coordinated interventions designed to improve and measure the appropriate use of antimicrobials by promoting the selection of the optimal antimicrobial drug regimen, dose, duration of therapy, and route of administration.<br>Reference: IDSA : Antimicrobial Stewardship, Infectious Diseases Society of America<br><br>After reading the definition, I have a better understanding of ASP	☐ Yes, a lot better<br>☐ Yes, a bit better<br>☐ No - I already had a good understanding<br>☐ I remain unsure what it means in practice
	Current ASP practices in community pharmacies	
10)	I provide the patient with clear messages on what side effects can be expected from the antibiotic.	☐ Do not practice at all<br>☐ Practice on some occasions<br>☐ Practice on most occasions<br>☐ Practice all the time<br>☐ No opinion
11)	I provide the patient with clear messages on what they should do if they experience side effects from the antibiotic.	☐ Do not practice at all<br>☐ Practice on some occasions<br>☐ Practice on most occasions<br>☐ Practice all the time<br>☐ No opinion
12)	I contact the prescriber if a prescription is for an antibiotic the patient is allergic to.	☐ Do not practice at all<br>☐ Practice on some occasions<br>☐ Practice on most occasions<br>☐ Practice all the time<br>☐ No opinion
13)	I contact the prescriber if the antibiotic dose/frequency is too high or too low.	☐ Do not practice at all<br>☐ Practice on some occasions<br>☐ Practice on most occasions<br>☐ Practice all the time<br>☐ No opinion
14)	I contact the prescriber if the prescription of an antibiotic involves a clinically significant drug interaction.	☐ Do not practice at all<br>☐ Practice on some occasions<br>☐ Practice on most occasions<br>☐ Practice all the time<br>☐ No opinion
15)	I contact the prescriber if I have reason to believe that the choice of antibiotic may not be optimal, in terms of the infection the patient is experiencing.	☐ Do not practice at all<br>☐ Practice on some occasions<br>☐ Practice on most occasions<br>☐ Practice all the time<br>☐ No opinion
16)	When a patient comes to me with symptoms of infection not needing antibiotics, I recommend OTC/self-care treatment.	☐ Do not practice at all<br>☐ Practice on some occasions<br>☐ Practice on most occasions<br>☐ Practice all the time<br>☐ No opinion

17) I refer a patient to a general practitioner when their symptoms are suggestive of an infection requiring an antibiotic and their symptoms have not improved from OTC/self-care treatment, or the severity of their infection appears to be more serious.

- ☐ Do not practice at all
- ☐ Practice on some occasions
- ☐ Practice on most occasions
- ☐ Practice all the time
- ☐ No opinion

18) When a patient is given an original prescription for an antibiotic that includes a refill, I provide advice when it would be appropriate to use that refill.

- ☐ Do not practice at all
- ☐ Practice on some occasions
- ☐ Practice on most occasions
- ☐ Practice all the time
- ☐ No opinion

19) When a patient presents a refill prescription for an antibiotic, I ask some questions to try and determine if it is appropriate for them to use that refill.

- ☐ Do not practice at all
- ☐ Practice on some occasions
- ☐ Practice on most occasions
- ☐ Practice all the time
- ☐ No opinion

20) Based on your professional experience, please provide any other comments, suggestions, or opinions about current practices of ASP in community pharmacies:

____

Perceived importance of ASP in community pharmacies

21) Community pharmacists can play an important role in the implementation of ASP.

- ☐ Strongly disagree
- ☐ Disagree
- ☐ Neither disagree nor agree
- ☐ Agree
- ☐ Strongly agree

22) ASP will reduce health care costs associated with infections.

- ☐ Strongly disagree
- ☐ Disagree
- ☐ Neither disagree nor agree
- ☐ Agree
- ☐ Strongly agree

23) Participation in ASP will enhance the public image of community pharmacists.

- ☐ Strongly disagree
- ☐ Disagree
- ☐ Neither disagree nor agree
- ☐ Agree
- ☐ Strongly agree

24) Participation in ASP will enhance the job satisfaction of community pharmacists.

- ☐ Strongly disagree
- ☐ Disagree
- ☐ Neither disagree nor agree
- ☐ Agree
- ☐ Strongly agree

25) Implementing ASP in community pharmacies will reduce inappropriate antibiotic use.

- ☐ Strongly disagree
- ☐ Disagree
- ☐ Neither disagree nor agree
- ☐ Agree
- ☐ Strongly agree

26) Please provide any other comments, suggestions, or opinions about your perception of ASP in community pharmacies:

____

Barriers to ASP implementation in community pharmacies

27) I do not have the required training to participate in ASP programs.

- ☐ Strongly disagree
- ☐ Disagree
- ☐ Neither disagree nor agree
- ☐ Agree
- ☐ Strongly agree

28) I do not have enough time to participate in ASP programs.

- ☐ Strongly disagree
- ☐ Disagree
- ☐ Neither disagree nor agree
- ☐ Agree
- ☐ Strongly agree

29) There aren't any standard guidelines for community pharmacists to implement ASP programs.

- ☐ Strongly disagree
- ☐ Disagree
- ☐ Neither disagree nor agree
- ☐ Agree
- ☐ Strongly agree

30) There is limited access to essential patient information, including clinical and laboratory data, to review the appropriateness of antibiotic prescriptions.

- ☐ Strongly disagree
- ☐ Disagree
- ☐ Neither disagree nor agree
- ☐ Agree
- ☐ Strongly agree

31) Medical providers are not receptive to pharmacists intervening on the choice of antibiotic.

- ☐ Strongly disagree
- ☐ Disagree
- ☐ Neither disagree nor agree
- ☐ Agree
- ☐ Strongly agree

32) Medical providers are not receptive to pharmacists intervening on the dose and dosage form of an antibiotic.

- ☐ Strongly disagree
- ☐ Disagree
- ☐ Neither disagree nor agree
- ☐ Agree
- ☐ Strongly agree

33) Medical providers are not receptive to pharmacists intervening on the duration of an antibiotic.

- ☐ Strongly disagree
- ☐ Disagree
- ☐ Neither disagree nor agree
- ☐ Agree
- ☐ Strongly agree

34) Patients always pushback or pressure pharmacists to give medications that does not seem appropriate.

- ☐ Strongly disagree
- ☐ Disagree
- ☐ Neither disagree nor agree
- ☐ Agree
- ☐ Strongly agree

35) Please provide any other comments, suggestions, or opinions which you believe are a major barrier in implementing ASP in community pharmacies:

____

Facilitators of ASP in community pharmacies

36) How helpful would you find the following: Increase provision of educational activities regarding ASP (e.g. workshops, webinars, lectures, etc.).

- ☐ Unhelpful
- ☐ Little helpful
- ☐ Somewhat helpful
- ☐ Most helpful
- ☐ No opinion

37) How helpful would you find the following: Public awareness initiatives (in electronic or print media) highlighting the role of community pharmacists in ASP.

- ☐ Unhelpful
- ☐ Little helpful
- ☐ Somewhat helpful
- ☐ Most helpful
- ☐ No opinion

38) How helpful would you find the following: Access to guidelines for common community infections.

- ☐ Unhelpful
- ☐ Little helpful
- ☐ Somewhat helpful
- ☐ Most helpful
- ☐ No opinion

39) How helpful would you find the following: Better collaboration with local medical providers practices.

- ☐ Unhelpful
- ☐ Little helpful
- ☐ Somewhat helpful
- ☐ Most helpful
- ☐ No opinion

40) How helpful would you find the following: Clarifications of the roles and responsibilities of pharmacists by regulatory and professional organizations (State Pharmacy Boards).

- ☐ Unhelpful
- ☐ Little helpful
- ☐ Somewhat helpful
- ☐ Most helpful
- ☐ No opinion

41) How helpful would you find the following: Monetary compensation for the time involved in ASP programs.

- ☐ Unhelpful
- ☐ Little helpful
- ☐ Somewhat helpful
- ☐ Most helpful
- ☐ No opinion

42) How helpful would you find the following: Better access to patient's clinical and laboratory data.

- ☐ Unhelpful
- ☐ Little helpful
- ☐ Somewhat helpful
- ☐ Most helpful
- ☐ No opinion

43) Please provide any other comments, suggestions, or opinions to support your role in quality use of antibiotics:

____
